# The significance of translation regulation in the stress response

**DOI:** 10.1186/1471-2164-14-588

**Published:** 2013-08-28

**Authors:** Flora Picard, Pascal Loubière, Laurence Girbal, Muriel Cocaign-Bousquet

**Affiliations:** 1 Université de Toulouse; INSA, UPS, INP; LISBP, 135 Avenue de Rangueil, F-31077, Toulouse, France; 2 INRA, UMR792 Ingénierie des Systèmes Biologiques et des Procédés, F-31400, Toulouse, France; 3 CNRS, UMR5504, F-31400, Toulouse, France

**Keywords:** Translational regulation, Stress, Bacterial adaptation, Gene expression regulation, Post-transcriptional regulation, Lactococcus lactis

## Abstract

**Background:**

The stress response in bacteria involves the multistage control of gene expression but is not entirely understood. To identify the translational response of bacteria in stress conditions and assess its contribution to the regulation of gene expression, the translational states of all mRNAs were compared under optimal growth condition and during nutrient (isoleucine) starvation.

**Results:**

A genome-scale study of the translational response to nutritional limitation was performed in the model bacterium *Lactococcus lactis*. Two measures were used to assess the translational status of each individual mRNA: the fraction engaged in translation (ribosome occupancy) and ribosome density (number of ribosomes per 100 nucleotides). Under isoleucine starvation, half of the mRNAs considered were translationally down-regulated mainly due to decreased ribosome density. This pattern concerned genes involved in growth-related functions such as translation, transcription, and the metabolism of fatty acids, phospholipids and bases, contributing to the slowdown of growth. Only 4% of the mRNAs were translationally up-regulated, mostly related to prophagic expression in response to stress. The remaining genes exhibited antagonistic regulations of the two markers of translation. Ribosome occupancy increased significantly for all the genes involved in the biosynthesis of isoleucine, although their ribosome density had decreased. The results revealed complex translational regulation of this pathway, essential to cope with isoleucine starvation.

To elucidate the regulation of global gene expression more generally, translational regulation was compared to transcriptional regulation under isoleucine starvation and to other post-transcriptional regulations related to mRNA degradation and mRNA dilution by growth. Translational regulation appeared to accentuate the effects of transcriptional changes for down-regulated growth-related functions under isoleucine starvation although mRNA stabilization and lower dilution by growth counterbalanced this effect.

**Conclusions:**

We show that the contribution of translational regulation to the control of gene expression is significant in the stress response. Post-transcriptional regulation is complex and not systematically co-directional with transcription regulation. Post-transcriptional regulation is important to the understanding of gene expression control.

## Background

The microbial cell response to environmental stress is complex. Multi-stage regulation at transcriptional and post-transcriptional levels contributes to reprogramming protein synthesis. Stress responses have been extensively studied at the transcript level; transcriptome changes generally involve increased expression of stress-defense genes and reduced expression of those encoding ribosomal proteins and all other growth-related systems [1,2]. As cells respond to stress, the proteome also changes [1-3] but most recent proteomics studies in microorganisms have reported poor correlation between transcriptome and proteome responses [4-9]. Therefore, there is presumably a translational response that contributes to adaptation to stress.

There have been few genome-wide studies of translation regulation in microorganisms. Both the translational rate and polysome size for most transcripts are lower in yeast under stress conditions than under normal conditions [10-12]. This response has been associated with the modulation of gene-specific mRNA translation efficiency to maintain and even increase the recruitment of ribosomes to mRNAs encoding proteins with functions essential for responding to stress. Co-directional changes at the levels of transcription and translation have been observed in yeast for most genes but not all, and some show antagonistic regulation at the mRNA and translation levels [9,12-14]. In bacteria, there has been no such comparison of translatome data between optimal growth and stress conditions; the translational response in bacteria to stress and its relative contribution to the regulation of gene expression are still to be determined. At low growth rate, Bremer and Dennis estimated that the total number of ribosomes in *Escherichia coli* would be only one tenth of that at high growth rate [15]. The consequence of the lower number of ribosomes on the control of the rate of translation is uncertain: the average rate of translation of bulk mRNA decreases at decreasing growth rate whereas an approximately constant rate of translation of *lacZ* mRNA is observed [16].

Amino acid assimilation is essential for optimal growth for most bacteria; this is particularly true for Lactic Acid Bacteria, most of which are auxotrophic for amino acids. A genome-wide analysis of the response of *Lactococcus lactis,* the model lactic acid bacterium, to progressive isoleucine starvation revealed an extensive transcriptional response [2]. It involves a gradual generalized reduction in the major physiological activities associated with a more specific positive response dedicated to coping with the imposed nutritional starvation. *L. lactis* does not have any stress-related alternative sigma factor [17] but several global regulatory mechanisms have been demonstrated to be involved in the regulation of this transcriptional response (*i.e*. growth rate-related mechanism, stringent response and the transcriptional regulator, CodY) [2]. Both the stringent response and the growth rate related regulation contributed at around 30% to the transcriptional response under isoleucine starvation. There are also changes in the proteome during isoleucine starvation [2]. However, systemic comparison of transcriptomic and proteomic data revealed only a weak correlation, suggesting that the regulation of translation has a major role in this stress response [4].

To describe the reprogramming of *L. lactis* gene expression associated with isoleucine starvation, we investigated the contribution of the translational response. First, we determined the detailed translational response of *L. lactis* to isoleucine starvation. Ribosome occupancy and ribosome density were compared between optimal growth and stress conditions for each individual transcript. The translatome profile in optimal growth conditions has already been reported [18]. Second, using a vertical integrative approach, we studied the role of the translation response in the global process of gene expression regulation under isoleucine starvation condition. In particular, we compared the influence of the regulation of translation with that of other post-transcriptional regulations, *i.e.* mRNA degradation (data also available in conditions of isoleucine starvation [19]) and mRNA dilution by growth.

## Results

### Polysome profile under isoleucine starvation

**Figure 1:**
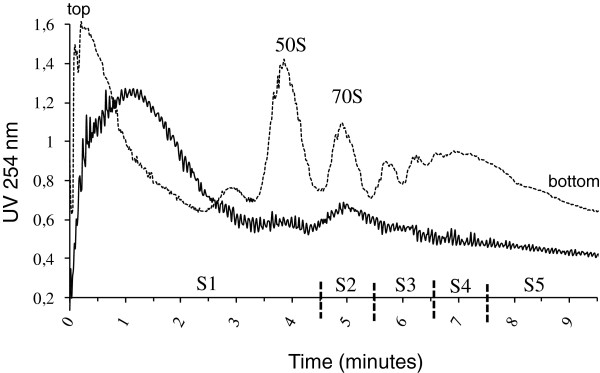
**Polysome profile in *L. lactis *cells under stress conditions (continuous line).** For comparison, the polysome profile previously observed in optimal growth conditions (dotted line) is also included [18]. Polysome complexes from cells grown under stress conditions were fractionated into five fractions: S1, elution time between 0 and 4.5 minutes; S2, elution time between 4.5 and 5.5 minutes; S3, elution time between 5.5 and 6.5 minutes; S4, elution time between 6.5 and 7.5 minutes; S5, elution time after 7.5 minutes.

The translatome profile was determined for *L. lactis* cells in conditions of isoleucine starvation (total exhaustion of isoleucine from the growth medium). In this stress condition, *L. lactis* grew constantly but very slowly: 0.05 h^-1^ to be compared to 0.88 h^-1^ in optimal growth conditions. Translation elongation was arrested and cells lyzed, and mRNA-ribosome complexes were separated according to the polysome size. Typical polysome profile and peak assignment under stress conditions are shown in Figure 1. In stress conditions, the polysomal profile was less clearly resolved than optimal growth conditions [18] and showed a broad initial peak of absorbance (during the first three minutes) corresponding to low molecular weight proteins confined in the upper layer of the sucrose density gradient. Elution fractions were pooled into five fractions named S1, S2, S3, S4 and S5 (see Methods). RNA concentrations in the first and last eluted fractions were lower in cells under stress than optimal growth conditions, leading to a fractionation into a total of five fractions instead of seven in optimal growth conditions [18].

Fraction S1 contained free mRNAs or mRNAs loaded with an incomplete ribosome; S2 contained mRNAs bound to one ribosome (monosome fraction). The number of ribosomes per transcript in the polysomal fractions S3, S4 and S5 has been estimated as previously [18] and the averages are in the ranges 1.4-2.9 (mean value 2.1), 2.9-5.1 (mean value 3.9), 5.1-15.1 (mean value 8.4), respectively. In stress conditions, the maximum number of ribosomes per transcript was 15 whereas it is 18 in optimal growth conditions [18]. The total RNA concentration in cells in conditions of isoleucine starvation was almost half that in optimal growth conditions (5.43 ± 0.50 and 9.01 ± 0.16 g.100 g^-1^ cells, respectively). Ribosomal RNAs are the major components of the total RNA population; therefore this suggests that there is a significant reduction of the total number of ribosomes and of the polysome size during stress. We checked that mRNA as a proportion of total RNA (estimated by summation of all microarray spot intensities) did not differ between optimal growth conditions and isoleucine starvation. The percentage of ribosomes engaged in translation, estimated by area integration of the absorbance of the polysomal profile as described previously [18], was also not significantly affected by isoleucine starvation: the mean values were 59 ± 3% in conditions of isoleucine starvation and 61 ± 2% in optimal growth conditions [18].

### Influence of isoleucine starvation on ribosome density and ribosome occupancy

**Figure 2:**
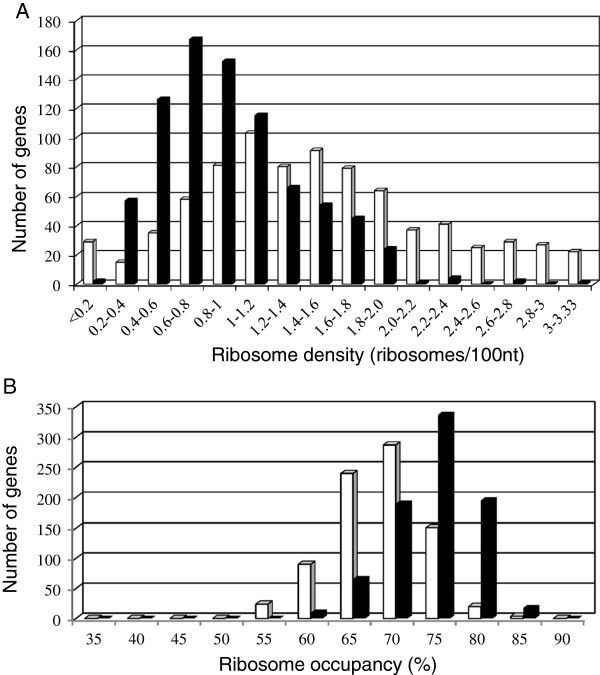
**Distribution of ribosome density and ribosome occupancy in stress conditions.** Ribosome density **(A)** and ribosome occupancy **(B)** were determined for 816 genes under stress (black bars). For comparison, the ribosome density and ribosome occupancy of the same genes in optimal growth conditions (white bars) are also included [18].

**Figure 3:**
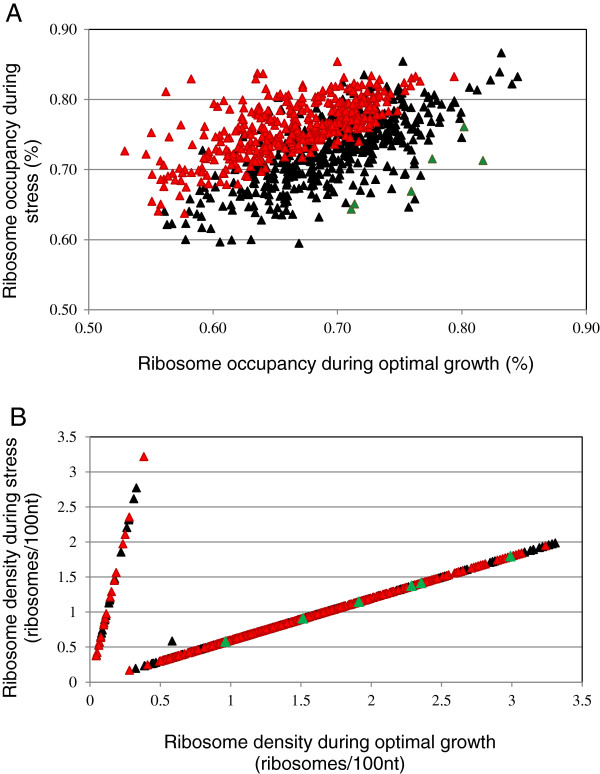
**Variation in ribosome occupancy and ribosome density under stress and optimal growth conditions. (A)** Ribosome occupancy: genes with a significantly higher ribosome occupancy in stress compared to optimal growth conditions are shown in red symbols (375 genes) and those with significantly lower ribosome occupancy are depicted in green (6 genes). Black symbols indicate genes (435 genes) with similar ribosome occupancy in stress and optimal growth conditions (Student test, p-value < 0.05). **(B)** Ribosome density: genes with higher ribosome density in stress than optimal growth conditions are indicated by the upper line (39 genes) while those with lower ribosome density are displayed by the lower line (776 genes). The isolated symbol is the only gene with a similar ribosome density in both stress and optimal growth conditions. The color code of the genes attributed based on ribosome occupancy in **A** was reused in **B**.

In *L. lactis* cells starved of isoleucine, the peak fraction corresponding to the most frequent number of ribosomes bound on each mRNA was identified for each of 1317 genes and the fractions in which it was found were identified with a 95% bootstrap confidence interval. Only 29 genes had a peak fraction overlapping two or three fractions. We therefore focused our analysis on the 1288 genes with a peak fraction confined to a single fraction. Most of these genes were heavily ribosome-loaded with the peak fraction in fraction S5, corresponding to 5.1-15.1 bound ribosomes. Only one gene (*yohD,* unknown function) had a peak fraction in the monosome fraction (S2). For each of the 1288 genes, ribosome density was calculated as the ratio of the ribosome number in its peak fraction to the coding sequence length. However, this calculation attributed ribosome densities above the maximal theoretical ribosome density (3.33 ribosomes/100 nucleotides) to 47 genes of the 1288 gene set. These 47 genes with aberrant ribosome densities were omitted from the subsequent analyses. Among 1241 remaining genes, ribosome density was obtained in both optimal growth and stress conditions for 816: the ribosome density ranged from 0.04 to 3.31 ribosomes per 100 nucleotides (Figure 2A). For cells in isoleucine starvation, the median ribosome density was 0.89 ribosomes per 100 nucleotides whereas it was 1.41 ribosomes per 100 nucleotides in optimal growth conditions [18]; indeed, the ribosome density was significantly lower in stress than in optimal growth conditions for 95% of the genes (Figure 3B, lower line of genes). The interpretation of ribosome density can vary according to the limiting step of translation. For genes exhibiting initiation-limited translation, high ribosome density is correlated with high translation levels, but for genes with elongation limitation, high density can result from ribosome congestion leading to low translation efficiency. The ribosome density far lower than the maximal theoretical density suggests that the translation of most mRNAs in stress conditions may be initiation–limited, as previously suggested in optimal growth conditions [18]. Further evidence for this was recently provided by kinetic modeling of bacterial translation from experimentally determined ribosome densities for *L. lactis* mRNAs in both conditions: translation was mainly initiation-limited for 89% and 69% of the genes in optimal growth and stress conditions, respectively [20]. Most *L. lactis* mRNAs are expected to be subject to initiation-limited translation, a decrease of the ribosome density in stress conditions presumably reflects less translation. Only 39 genes showed increased ribosome density in conditions of isoleucine starvation (Figure 3B, upper line of genes): the corresponding peak fractions moved from the monosome fraction under optimal growth conditions to the polysomal fractions under stress conditions. This set of 39 genes included several prophage genes (*pi116, pi218, pi228, pi231, pi245, pi331*, *ps207* and *ps214*).

Ribosome occupancy was calculated for each transcript species as a measure of efficiency of translation initiation; values for each mRNA were compared between stress and optimal growth conditions. In stress conditions, the distribution of ribosome occupancy was shifted toward higher values (Figure 2B). For the set of 816 selected genes (see above), the median ribosome occupancy value for their mRNAs was 74% during isoleucine starvation and 68% in optimal growth conditions [18]; there was a statistically significant difference of ribosome occupancy between the two conditions for 381 genes (46%). Ribosome occupancy in stress conditions was higher for 375 mRNAs and lower for only six (*accB* and *fabG1* related to fatty acid biosynthesis*, ptnD* and *ypcG* involved in sugar transport and *ychG* and *yvcA* with unknown functions) (Figure 3A).

### Impact of isoleucine starvation on gene translation regulation

To examine the consequences of isoleucine starvation on the regulation of gene translation, we studied the effects of stress on both ribosome occupancy and ribosome density: the differences in both ribosome occupancy and ribosome density between the two conditions were analyzed for each mRNA from the set of 816 genes. We plotted the variation in ribosome density under stress and optimal growth conditions (Figure 3B). The upper line indicated genes moving from the monosome fraction in optimal growth conditions to an average 8.4 ribosomes per transcript in stress conditions. One gene *yohD* remained in the monosome fraction in both conditions. The lower line displayed genes with a higher average number of ribosomes per transcript in optimal growth conditions than in stress conditions, 14 against 8.4. Black, red and green symbols indicate respectively genes with similar, higher and lower ribosome occupancy in stress than optimal growth conditions. The resolution of the translatome experiment under stress conditions was lower than in optimal growth conditions (5 elution fractions instead of 7). Therefore, part of the variability of the translational response in stress conditions could be hidden in our ribosome density comparison and thus not displayed in Figure 3B. Nevertheless, the observation of linear profiles suggests that except for one gene, most genes can be clustered into two classes of “density regulation” reflecting variation in translation elongation under isoleucine starvation may be related to isoleucine codon bias or other sequence specificities.

We identified only one gene *yohD* for which neither measure was significantly modified. *yohD* was thus considered to not be translationally regulated by stress conditions. There were 39 genes for which translation was up-regulated under stress conditions: for 19 genes, both translational measures were higher (Figure 3B, red genes in the upper gene line) and for 20 genes, the ribosome density was higher and the ribosome occupancy was not significantly affected (Figure 3B, black genes in the upper gene line). The translation of the mRNAs for 420 genes was down-regulated under stress conditions: the six genes described above for which both translational measures were lower (Figure 3B, green genes in the lower gene line); and 414 genes for which the mRNA showed lower ribosome density and similar ribosome occupancy (Figure 3B, black genes in the lower gene line). For a surprisingly large number of genes (356 genes), the mRNA showed a lower ribosome density but a higher ribosome occupancy in stress than optimal growth conditions (Figures 3B, red genes in the lower gene line). Thus, the translation of the mRNAs of these genes appears to be subject to two antagonistic regulations.

Functional analysis based on category enrichment tests showed a relationship between translation regulation and gene function. Prophage genes (including genes *pi116, pi218, pi228, pi231, pi245, pi331*, *ps207* and *ps214)* were over-represented in the translationally up-regulated group indicating that the mRNAs of these genes were more translated in stress conditions. Genes involved in growth-related functions were enriched in the translationally down-regulated group: translation [ribosomal genes encoding proteins involved in protein synthesis (*fmt, gatB, ksgA, miaA, prmA, rplI, rpsC, truA, truB, ycjD* and *yhdC*), several translational factors (*efp, Frr, fusA, lepA* and *prfB*) and amino acyl tRNA synthetases (*alaS, aspS, hisS, leuS, lysS, pheS, pheT, proS, thrS* and *valS*)], transcription [genes related to RNA polymerase (*rpoB, rpoC* and *rpoE*), to pseudouridine synthase (*rluA, rluB, rluC* and *rluD*) and to RNA degradation *(pnpA* and *vacB1*)], base metabolism [genes of the purine (*guaC, purB, purC, purD, purE, purF, purH, purK, purl, purM* and *purN*) and pyrimidine (*carA, carB, pydB, pyrB, pyrC, pyrF* and *pyrZ*) biosynthetic pathways] and fatty acid and phospholipid metabolism (*accB, accC, accD, fabF, fabG1, fabZ2, fadD, lplL, plsX* and *yscE*). These growth-related functions were thus down-regulated at the translational level during isoleucine starvation, and are presumably responsible for the decreasing growth rate during the stress. The complete *leu-ilv* operon involved in the biosynthesis of isoleucine was subject to antagonistic translational regulation: ribosome occupancy was higher and ribosome density lower in stress than optimal growth conditions for all the seven transcripts detected (*leuB, leuC, leuD, ilvB, ilvC, ilvD* and *ilvN;* targets of *ilvA* and *leuA* were not available on the array).

### Different regulatory patterns of gene expression for adaptation to isoleucine starvation

Translation is one of the downstream processes at which gene expression can be controlled. Indeed, protein production is dependent on the regulation of translation (related to ribosome density and ribosome occupancy) and also on the level of mRNA concentration. The concentration of any particular mRNA depends on the balance between transcription rate, degradation rate and rate of dilution by growth. We investigated the relative importance of translation regulation in this multilevel network during isoleucine starvation. We calculated ratios of the transcription, mRNA degradation and dilution rate constants between stress and optimal growth conditions and compared these ratios with those for ribosome occupancy and ribosome density (Additional file 1: Table S1).

In the stress condition studied, rate constants of transcription, mRNA degradation and dilution by growth were all substantially lower than in optimal growth conditions (at least 85% of the rate constant ratios were lower than 0.9). Ribosome densities were significantly lower in stress conditions (96% of the ratios ≤ 0.6) whereas ribosome occupancy behavior was diverse (higher (ratios above 1.1) for 42% of the mRNAs and lower for 10%). Isoleucine starvation thus resulted in a general trend of reduced mRNA production by transcription, but slower mRNA loss, both degradation and dilution by growth being slower.

**Figure 4:**
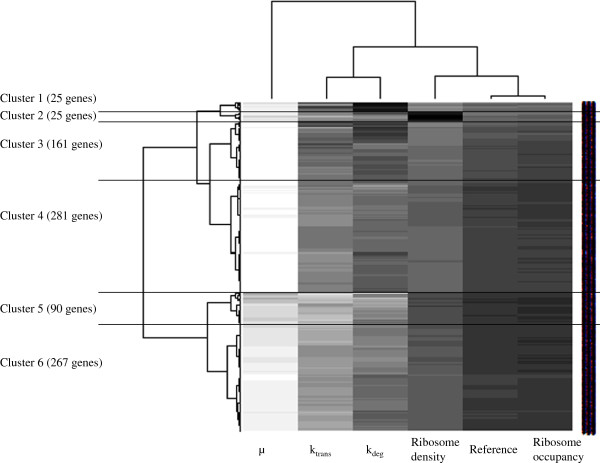
**Hierarchical clustering of genes based on their pattern of expression regulation.** 849 genes were clustered using ratios between stress and optimal growth conditions of rate constants (transcription *k*_*trans*_, degradation *k*_*deg*_ and dilution rate *μ*) and of translational measures (ribosome density and ribosome occupancy). The column ‘reference’ corresponds to a ratio of 1. For each gene, colors darker and lighter than the reference indicate ratios higher and lower than 1, respectively.

These various ratios were used for hierarchical clustering of the genes (Figure 4). Six gene clusters were thereby defined with different patterns of regulation of gene expression. In clusters 4 to 6, the general regulatory pattern described above was respected, and the differences between clusters mostly involved the magnitude of the difference of the transcription rate constant. Patterns in clusters 1, 2 and 3 differed from the general regulatory pattern. Clusters 1 and 3 consisted of genes for which the mRNA degradation rate constant was higher in stress than optimal growth conditions. The regulation of the expression of genes in cluster 2 involved a much higher ribosome density under stress than optimal growth conditions.

At the functional level, only the prophage category was over-represented in the groups with atypical patterns: 20 prophage genes (Additional file 1: Table S1) were found in clusters 1 or 3. This is consistent with the destabilization of their mRNA exhibiting half-lives from more than 47 minutes under optimal growth conditions to an average of 17 minutes under isoleucine starvation [19]. Average mRNA half-lives were 6.2 minutes in optimal growth conditions and 12.1 minutes in conditions of isoleucine starvation, so these prophage mRNAs can still be considered to be stable transcripts. Prophage genes *pi116, pi228* and *pi245* were classified into cluster 2, in agreement with their presence in the group of genes with enhanced ribosome density under isoleucine starvation.

The isoleucine starvation response has been studied previously by transcriptome analysis: the concentration of mRNAs associated with growth-related functions was demonstrated to decrease whereas that of mRNAs of the isoleucine biosynthetic pathway specifically increased [2]. Our results (Additional file 1: Table S1) indicated that the regulation of *ilvB, ilvC, leuC* and *leuD* gene expression (classified in clusters 4 or 5) followed the general trend: the higher mRNA levels during isoleucine starvation were not related to an increase of the transcription rate but to a decrease of the transcription rate counteracted by mRNA stabilization and reduced dilution by growth. The higher mRNA levels for *ilvD, ilvN* and *leuB* (genes classified in clusters 1 and 3), were associated with increased transcription rates, and for *ilvN* and *leuB* with a concomitant antagonistic mRNA destabilization. Genes encoding proteins with the growth-related functions, translation, transcription, fatty acid and phospholipid metabolism and base metabolism were almost exclusively classified in clusters 4, 5 and 6, and displayed the general trend of gene expression regulation: their lower mRNA levels under isoleucine starvation corresponded to lower transcription rate with only small contributions from the opposite influences of mRNA stabilization and reduced dilution by (slow) growth.

### Influence of the regulatory pattern of gene expression on protein profiles under isoleucine starvation

We tested whether the patterns of regulation of gene expression, corresponding to the six clusters, were associated with different protein expression profiles. Ratios of protein concentration between stress and optimal growth conditions were calculated for the products of 107 genes, from available proteomics analyses [2]. These ratios were compared to the clustering of the regulation of the expression of the corresponding gene. No particular protein ratio could be associated to any of the six clusters, and the protein ratios did not allow the different clusters to be discriminated (Additional file 2: Figure S1). This indicates the absence of any direct relationship between a type of regulatory pattern of gene expression and the resulting regulation of protein levels under isoleucine starvation.

We analyzed, in more detail, proteins related to translation, and in particular ribosomal proteins (Fmt, GatB, RplI, RplV, RpsC and RpsS)*,* translational factors (Frr, FusA, PrfA and PrfC*)* and amino acyl tRNA synthetases (AspS, LeuS, LysS, MetS, PheT, ProS, ThrS and TrpS). The genes for RpsS, PepP and PrfC were in clusters 1 or 3, and the genes of all the other proteins were in clusters 4 to 6 displaying the general trend of gene expression regulation. Half of these proteins were more abundant in isoleucine starvation than optimal growth conditions, whereas the other half were less abundant. Similarly, other growth-related proteins (five proteins of fatty acid and phospholipid metabolism, 13 of base metabolism and three of transcription), also encoded by genes in clusters 4 to 6, were either more or less abundant under stress; indeed, in this group of growth-related proteins, some of the expression ratios were extreme (0.44 for the reductase FabG1 of fatty acid biosynthesis and above 20 for the synthetase PurM of purine biosynthesis). Two dehydratases of the isoleucine biosynthesis pathway, IlvD and LeuC, encoded by genes in cluster 3 and cluster 5, were significantly more abundant in isoleucine starvation than optimal growth conditions (ratios of 4.3 and 2.8, respectively).

## Discussion

Isoleucine starvation imposes stress on *L. lactis* inducing a global physiological response consistent with slower growth and a specific response to facilitate the *de novo* synthesis of isoleucine [2]. In this work, we compared the translational status of numerous individual mRNAs in *L. lactis* cells under optimal growth conditions to that in cells subjected to isoleucine starvation (growing at a slow residual growth rate). Under stress conditions, 40% of the total ribosomal content and 26% of the total transcript content were not involved in mRNA-ribosome complexes. The limitation of translation by the initiation step of most of the genes could reflect the difficulty that mRNA and ribosome hit each other, particularly if different spatial distributions of mRNAs and ribosomes is assumed [21]. For each mRNA, the translational status was characterized by two measures: ribosome occupancy and ribosome density. Both measures were available for 816 genes under stress. For all these genes except one, one or both translational measures was significantly affected by the stress, and thus the gene expression was subject to translational regulation. Therefore, we report that the translational regulation in *L. lactis* has a major influence on gene expression.

The translational response of *L. lactis* under isoleucine starvation consisted of a general decrease of ribosome density for most of the genes and a lower maximal size of polysome complexes. Amino acid starvation of yeast has been reported to result in smaller numbers of loaded ribosomes [22]. A decline in the ribosome number appears to be a global response, indiscriminately affecting the different mRNA species with only few exceptions. Under a low availability of an amino acid, higher density could result from ribosome congestion near the codons of this amino acid when translation is elongation-limited by a low ratio of charged to uncharged tRNA [15]. We checked the isoleucine codon content of genes with up-regulated ribosome density under isoleucine depletion. Their coding sequence was not enriched in isoleucine codons compared to the rest of the gene population. Therefore elongation limitation near isoleucine codon is unlikely to explain the up-regulation of ribosome density. Prophage genes were over-represented in the set of 4% of genes that were translationally up-regulated, with enhanced ribosome density. Increased ribosome density and also mRNA destabilization (the atypical clusters 1, 2 and 3 were enriched in prophage genes) contributed to the atypical profile of the regulation of expression under isoleucine starvation. Prophage induction is of technological relevance, particularly for cheese-making, because it provokes *L. lactis* lysis and is responsible for cheese ripening [23]. The lower ribosome density in *L. lactis* cells under amino acid starvation was related to a smaller total number of ribosomes as reflected by the lower rRNA content in stressed cells. The stringent response activated under amino acid depletion is well-known to negatively regulate rRNA synthesis via (p)ppGpp [24,25]. The mRNA to rRNA ratio and the fraction of ribosomes involved in translation did not differ significantly between stress and optimal growth conditions. This is in agreement with reports correlating ribosome biogenesis to the growth rate in microorganisms, and with the observed constant percentage of translating ribosomes at different growth rates [15,26]. Nevertheless, ribosome activity could differ between stress and optimal growth conditions, since we can suspect that, when the stringent response is activated, only part of the ribosomes is efficient for translation due to limitation by the extent of tRNA charging.

The translation machinery is complex and composed of numerous ribosomal proteins, translation factors and amino acyl tRNA synthetases. We have demonstrated here that these genes were down-regulated, at the translational level, with decreased ribosome density, in stress conditions. The regulation of the expression of these genes during isoleucine starvation followed a general trend: reduced transcription rate counterbalanced by mRNA stabilization and slower mRNA dilution by growth. The negative co-regulation of transcription and translation for genes of the translation machinery during the stress response was thus clearly demonstrated; however, there were also concomitant antagonistic effects due to mRNA stabilization and reduced dilution by growth. Negative co-directional transcriptional and translational controls have been described in yeast for ribosomal protein expression in stress conditions [11]. However, in this case, the term “transcriptional” was referring to mRNA level regulation without discriminating between true control of mRNA production via transcription and the effects of mRNA consumption via degradation and dilution.

The response of ribosome occupancy during isoleucine starvation was less general than that of ribosome density; for more than half of the genes ribosome occupancy was unaffected. For growth-related processes, such as transcription, fatty acid and phospholipid metabolism and base metabolism, the lower ribosome density was associated with unchanged ribosome occupancy leading to translationally down-regulated mRNAs. The slowdown of growth of *L. lactis* under isoleucine starvation was related to the negative regulation at the translational level of these functions, which was accentuated by their lower transcription rate. Note that mRNA stabilization and less dilution due to cell growth of these mRNA molecules were acting together in the opposite direction. The balance between these different regulations led to a large range of protein concentration changes.

For most of the genes for which the ribosome occupancy of the mRNA differed between optimal growth and stress conditions, ribosome occupancy was higher (for 375 genes) in stress conditions; it was significantly lower for a small number of genes. Ribosome occupancy thus appeared to be responsible for the fine tuning of translation. By counterbalancing the negative effect of decreased ribosome density, regulation of ribosome occupancy allowed specific translational patterns. For example, genes of the *leu-ilv* operon involved in isoleucine biosynthesis were classified in the group of antagonistic translational regulations, with decreased ribosome density but increased ribosome occupancy in stress for these genes, there were also opposite regulations of transcription and mRNA stability (lower transcription rate but increased mRNA stability and vice versa). Therefore, *de novo* synthesis of isoleucine was under a complex set of controls comprising antagonistic regulations of both the concentration and translation of mRNA. This complex regulation network led to major protein re-organizations, and under stress the largest changes in protein abundance were observed for the IlvD and LeuC proteins involved in this pathway.

## Conclusions

We described the contribution of translational changes in the global process of the regulation of gene expression under isoleucine starvation. The regulations of mRNA translation, degradation and dilution by growth during isoleucine starvation were demonstrated to be either antagonistic or co-directional relative to transcription. Consequently, post-transcriptional regulation contributed actively to controlling gene expression during adaptation to stress. For a better understanding of protein concentration changes under stress conditions, this regulatory layer should be included in considerations of the multilevel regulation of gene expression. Although data for protein concentration changes were available for more than one hundred proteins in our study, we could not identify a direct link between the pattern of the regulation of gene expression and protein levels. Further investigation of protein concentrations and turn-over will be required for further elucidation of the control of protein abundance in *L. lactis* in response to stress conditions.

## Methods

### Organism and growth conditions

*L. lactis* subsp. *lactis* IL1403 was grown under anaerobic conditions in batch cultures at 30°C, pH 6.6 and 250 rpm, in a modified chemically defined medium in which the concentration of branched amino acids was only one tenth that in the standard medium ([isoleucine] = 150 μM, [leucine] = 360 μM, [valine] = 280 μM), as previously described [2]. Isoleucine starvation conditions started after isoleucine exhaustion at 3.66 h of culture [2].

### Polysomal RNA preparation

After 3 h of amino acid starvation corresponding to 6.66 h of culture (isoleucine starvation conditions), translation elongation was arrested by adding 100 mg/ml chloramphenicol and cells were collected on ice. Cells were harvested, resuspended in lysis buffer and washed twice [18]. Cell disruption using glass beads, size fractionation of mRNA-ribosome complexes on sucrose gradient and total RNA extraction were processed as described previously [18]. To obtain the 5 μg samples of total RNA required for transcriptomic analysis, the fractions eluted between 0 and 4.5 minutes, corresponding to free mRNAs or mRNAs loaded with an incomplete ribosome were pooled (fraction S1). The fractions eluted after 7.5 minutes were pooled (S5) and corresponded to more heavily ribosome-loaded mRNAs; three fractions were collected between 4.5 and 7.5 min (S2, S3 and S4). Each fraction was analyzed for 16S and 23S rRNA and peaks assigned for ribosomal subunits, and monosomal and polysomal complexes (data not shown). The five fractions, S1 to S5, were hybridized to the corresponding microarrays. An aliquot of cell-free extract containing unfractionated total RNA was used in parallel as a reference sample (named S0). These experiments were repeated with five independent cultures.

### Transcriptomic analysis of polysomal fractions and normalization

For signal normalization and modeling, the free statistical software R (http://www.r-project.org/) was used. Gene expression was measured using nylon arrays containing PCR fragments (Eurogentec®) for 1948 genes of *L. lactis* IL1403. The PCR fragments for each gene were spotted twice on the microarrays. Microarray spotting and analytical support were provided by the Biochips Platform (Genopole Toulouse, France). An aliquot of 5 μg of total RNA from each fraction (S0 to S5) was used for retrotranscription. RNA was quantified at 260 nm with Nanodrop (Thermo Scientific®). RNA quality was checked on Agilent 2100 Bioanalyzer (Agilent technologies®). Synthesis of radiolabeled cDNA, nylon array hybridization and washing were carried out as described previously [27]. Microarrays (from S0 to S5) were exposed to a phospho-imager screen for eleven days and scanned with a phospho-fluoroimager (Storm 860, Molecular Dynamics®). S0 to S5 fractions were prepared from five independent cultures, and thus five series of six microarrays were obtained. For each gene, the mean of the two spot intensities after subtraction of the background value was calculated. For each microarray, a cutoff value was defined as the mean intensity of the “empty” spots plus one standard deviation as previously described [27]. Genes presenting a mean intensity above the cutoff value on one of the six microarrays were selected for further analysis: 1709 genes were selected.

For each microarray series, all normalization steps including intra-series and inter-series normalization, correction of intensity values to the total RNA quantity and their centering reduction were as previously described [18].

### Calculations of translatome variables

For the 1709 genes with signal intensities above the cutoff values, two translatome variables were calculated: ribosome occupancy and ribosome density.

For each gene, ribosome occupancy is the percentage of its mRNA copies engaged in translation. It is calculated by summing the proportions of its mRNAs in fractions S2 to S5 [18]; mRNA molecules in fraction S1 were free or associated with an incomplete ribosome, so these mRNAs were not considered to be engaged in translation. For each gene, the five ribosome occupancy values obtained from the five independent series were averaged.

For each gene, the peak fraction corresponds to the highest mRNA proportion within fractions S2 to S5 containing mRNA engaged in translation. The peak fraction was determined by a bootstrap method on residuals [18]. Ten thousand iterations were performed and the relative frequency of the highest mRNA proportion was calculated with a confidence interval fixed at 95%. When the 95% bootstrap confidence interval was not confined to a single fraction, the definition of the peak fraction was widened from only one to two (or more) adjacent fractions and the search for the maximum was started again. In the set of 1709 genes, a peak fraction was assigned for 1317 genes: 1288 genes had a peak contained in a single fraction, 11 genes had a peak which overlapped two adjacent fractions, and 18 genes were found with a peak extending over three adjacent fractions. For each of the 1288 genes with their peak in a single fraction, we calculated the ribosome density as the number of bound ribosomes in the peak fraction normalized to the transcript length defined as the coding sequence length rather than the transcript length (which was not available).

Ribosome density and ribosome occupancy values are reported in Additional file 3: Table S2.

### Enrichment analysis and clustering

For various gene subsets, statistical tests were performed to assess the enrichment of genes having a characteristic of interest [18]. The p-value was calculated using R software and p-values lower than 0.05 were considered to indicate significant enrichment.

To examine the relative importance of the regulation of translation during isoleucine starvation, we calculated the transcription, mRNA degradation and dilution rate constants as follows. The mRNA degradation rate constant *k*_*deg*_ was obtained from half-life data:

(1) $${}_{k_{\deg}}=\frac{\ln2}{t_{1/2}}$$

with t_½_ values as determined after rifampicin addition to cultures in optimal growth and stress conditions [19]. The dilution rate constant corresponds to the growth rate *μ:*

(2) $$\mu =\frac{1}{X}\frac{\mathrm{dX}}{\mathrm{dt}}$$

where *X* is the biomass concentration. In optimal growth and stress conditions, growth rates were constant: 0.88 h^-1^ and 0.05 h^-1^, respectively. Assuming that a (pseudo)steady-state was established, the transcription rate *V*_*T*_ of a gene is the sum of the degradation and dilution rates, and the transcription rate constant *k*_*trans*_ can be calculated with the following equation:

(3) $$\begin{array}{ll} \;V_{T}= & \;k_{\mathrm{trans}}.\mathrm{genecopienumber}\\ & =k_{\deg}.\left[\mathrm{mRNA}\right]+\mu .\left[\mathrm{mRNA}\right] \end{array}$$

where *genecopienumber* is the number of copies of the gene in the cell. The numbers of gene copies were assumed to be constant in each optimal growth and stress conditions. The values used for mRNA concentrations in *L. lactis* cells under optimal growth *[mRNA]*^*optimal*^ and stress *[mRNA]*^*stress*^ conditions were those published previously in [2]. Hierarchical clustering of genes with similar pattern of expression regulation was obtained (ward method in R software) by comparing the ratio values of rate constants (transcription *k*_*trans*_, degradation *k*_*deg*_ and dilution rate *μ*) and of translational parameters (ribosome density and ribosome occupancy) between stress and optimal growth conditions. A reference ratio of 1 was introduced into the clustering to facilitate visualization of ratios higher or lower than 1 by color scaling.

## Competing interests

The authors declare that they have no competing interests.

## Authors’ contributions

FP, PL, MC-B, LG: conception, analysis of the data, drafting of the manuscript; FP: experimental work and data acquisition; FP, LG, MC–B: statistical data treatment. All authors read and approved the final manuscript.

## Supplementary Material

Additional file 1: Table S1 Ratios of rate constants, ribosome density and ribosome occupancy between optimal growth and stress conditions. Gene cluster and functional category (according to [17]) are also specified. Click here for file

Additional file 2: Figure S1 Protein concentration ratios between stress and optimal growth conditions, when available in [2]. The dotted line represents a ratio of 1. Click here for file

Additional file 3: Table S2 Ribosome density and ribosome occupancy values in *L. lactis* cells grown under isoleucine starvation. Click here for file
