# Supplementary figures and images for: The significance of translation regulation in the stress response
Source: BMC Genomics. 2013 Aug 28;14:588. doi: 10.1186/1471-2164-14-588 (PMC3765724; doi:10.1186/1471-2164-14-588)

## Slide 1
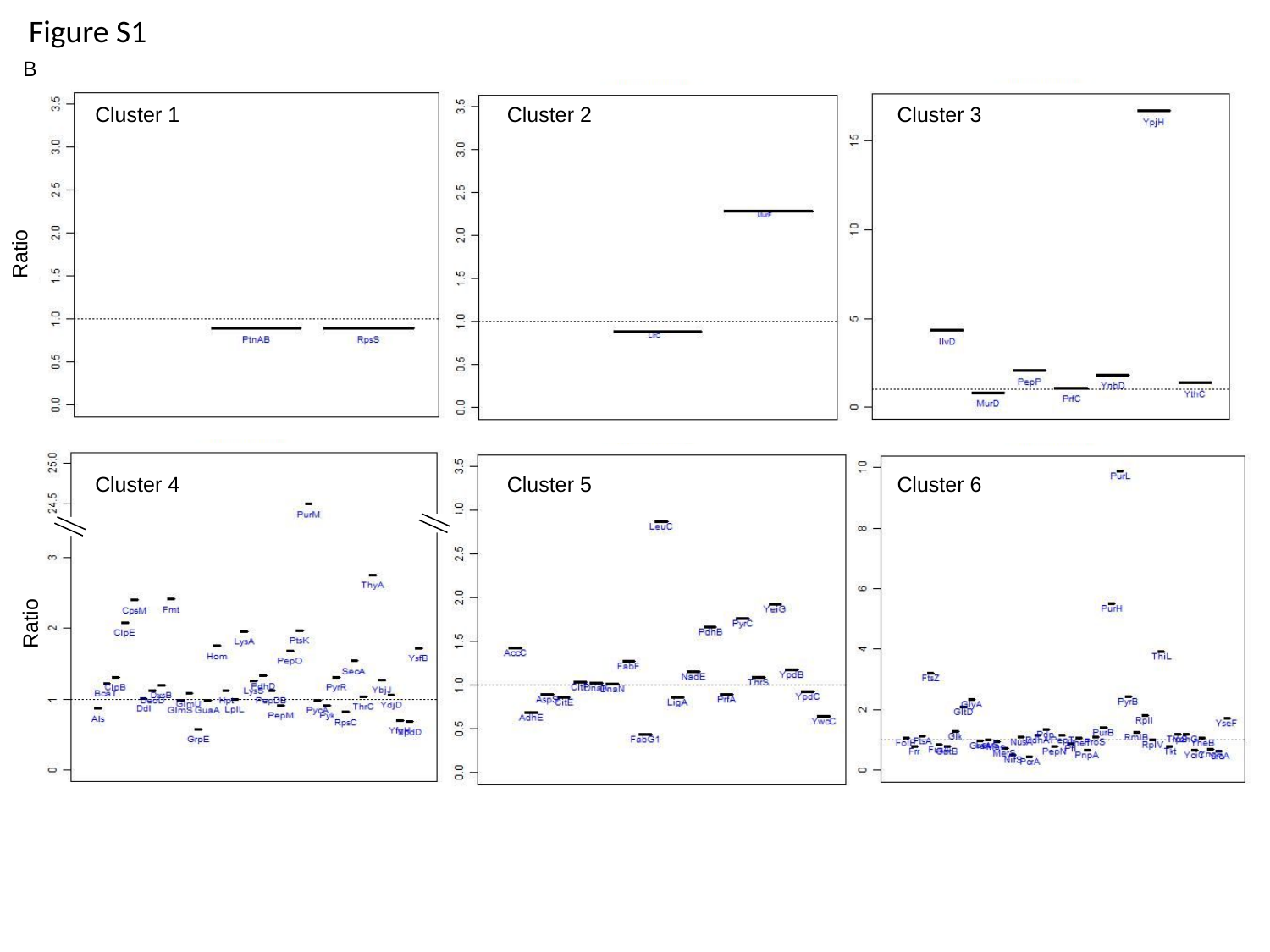

Figure S1
B
Cluster 1
Cluster 2
Cluster 3
Ratio
Cluster 4
Cluster 5
Cluster 6
Ratio

Supplement: Additional file 2: Figure S1 — Protein concentration ratios between stress and optimal growth conditions, when available in [2]. The dotted line represents a ratio of 1. [file 1471-2164-14-588-S2.pptx]
